# Glycoprotein B Antibodies Completely Neutralize EBV Infection of B Cells

**DOI:** 10.3389/fimmu.2022.920467

**Published:** 2022-05-27

**Authors:** Junping Hong, Dongmei Wei, Ling Zhong, Qian Wu, Kaiyun Chen, Wanlin Zhang, Yanbo Yang, Junyu Chen, Ningshao Xia, Xiao Zhang, Yixin Chen

**Affiliations:** ^1^State Key Laboratory of Molecular Vaccinology and Molecular Diagnostics, National Institute of Diagnostics and Vaccine Development in Infectious Diseases, School of Life Sciences, School of Public Health, Xiamen University, Xiamen, China; ^2^State Key Laboratory of Oncology in South China, Collaborative Innovation Center for Cancer Medicine, Guangdong Key Laboratory of Nasopharyngeal Carcinoma Diagnosis and Therapy, Sun Yat-sen University Cancer Center, Guangzhou, China

**Keywords:** Epstein–Barr virus, glycoprotein B, neutralizing antibodies, rhesus lymphocryptovirus, membrane fusion

## Abstract

The Epstein–Barr virus (EBV) is the first reported oncogenic herpesvirus that establishes persistent infection in B lymphocytes in 95% of adults worldwide. Glycoprotein B (gB) plays a predominant role in the fusion of the viral envelope with the host cell membrane. Hence, it is of great significance to isolate gB-specific fusion-inhibiting neutralizing antibodies (NAbs). AMMO5 is the only gB NAb but fails to antagonize B-cell infection. It is essential to isolate potent NAbs that can completely block EBV infection of B cells. Using hybridoma technology and neutralization assay, we isolate two gB NAbs 8A9 and 8C12 that are capable of completely neutralizing B-cell infection *in vitro*. In addition, 8A9 shows cross-reactivity with rhesus lymphocryptovirus (rhLCV) gB. Competitive binding experiments demonstrate that 8A9 and 8C12 recognize novel epitopes that are different from the AMMO5 epitope. The epitopes of 8A9 and 8C12 are mapped to gB D-II, and the AMMO5 epitope is located precisely at gB aa 410–419. We find that 8A9 and 8C12 significantly inhibit gB-derived membrane fusion using a virus-free fusion assay. In summary, this study identifies two gB-specific NAbs that potently block EBV infection of B cells. Our work highlights the importance of gB D-II as a predominant neutralizing epitope, and aids in the rational design of therapeutics or vaccines based on gB.

## Introduction

The Epstein–Barr virus (EBV) is a member of the γ-herpesvirus family, which establishes a lifelong infection in approximately 95% of human adults worldwide (1). As the first identified oncogenic virus in humans, EBV is included as a group 1 carcinogen by the World Health Organization (2, 3). EBV has a close association with carcinogenesis, and is linked to 1.5% of all cases of human malignances worldwide (4, 5). EBV infection is related to numerous tumors, including nasopharyngeal carcinoma (NPC), gastric cancer (GC), Hodgkin’s lymphoma (HL), and Burkitt’s lymphoma (BL) (6, 7). EBV-attributed cancers account for 200,000 new cases of malignancies and 140,000 deaths each year (8). In addition, EBV is also related to a number of non-malignant diseases such as infectious mononucleosis (IM), chronic active EBV infection (CAEBV), multiple sclerosis (MS), and EBV-associated hemophagocytic lymphohistiocytosis (EBV-HLH) (9–11). However, there are no available prophylactic vaccines or therapeutics for EBV (12).

Similar to the other herpesviruses, EBV infection is a complex multistep process that requires the involvement of multiple viral glycoproteins (13). Typically, EBV shows dual tropism and mainly infects epithelial cells and B cells, which can result in malignancies (14–17). EBV entry into distinct cell types requires different glycoproteins. Glycoproteins gp350, gp42, gH/gL heterodimers and gB are needed during the infection of B cells (18). EBV gp350 plays an important role in B-cell infection by increasing attachment to CR2, but is not actually needed; gp350-knockout EBV can still infect B cells to a lesser extent, though (19). Glycoprotein BMRF2, gH/gL, and gB are involved in the entry to epithelial cells (20, 21). gH/gL and gB participate in the infection of two cell types, and together comprise the fusion machinery of EBV (22, 23). gB is the viral fusion protein and the core component of the fusion machinery (18, 24, 25). Among the envelope glycoproteins of herpesvirus, gB is the most highly conserved glycoprotein in sequence and structure (26). In addition to being a fusogen, gB is also indispensable for the maturation of EBV virions, and egress of virus from the nucleus (27). Moreover, gB is also essential for viral replication, and EBV lacking the gene of gB cannot be produced (28, 29). EBV virions containing higher amount of gB show higher infectious efficiency (30). Taken together, gB is indispensable for viral maturation, egress, and especially viral entry into host cells.

EBV gB is the product of the BALF4 open reading frame (ORF) and categorized as a class III viral membrane fusion protein (30–32). Like other fusion proteins, gB tends to change from a metastable prefusion state to a highly stable postfusion state, which is irreversible (33, 34). The structure of the postfusion gB ectodomain has been solved using x-ray crystallography, and the crystal structure shows that trimeric gB adopts an elongated rod-like shape, which can be divided into 5 domains (26). EBV gB is reported to directly interact with the entry factor, neuropilin 1 (NRP1) during the infection process of nasopharyngeal epithelial cells (35). Being the fusogen, gB is the target for neutralizing antibodies (NAbs) and a vaccine candidate for herpesvirus (36–38). A number of potent NAbs targeting herpesvirus gB have been reported, and some of them are in the preclinical or clinical stages (39). Various herpesvirus vaccines based on gB show potent protection against viral infection and diseases (40–42). EBV gB is capable of inducing a neutralizing humoral response (43–45). The sera from trimeric gB-immunized rabbits provide efficient passive immune protection against lethal EBV challenge in a humanized mouse model, which shows better protective efficiency than that of gH/gL (43). Nevertheless, only a single EBV gB MAb, CL55, that is non-neutralizing, has been reported in a long time (46, 47). The human-derived antibody, AMMO5, is the first NAb targeting EBV gB (48). However, AMMO5 can only partially neutralize EBV infection of B cells, and the neutralizing epitope recognized by AMMO5 remains unknown. It is of great importance to generate potent gB NAbs that are able to completely neutralize B-cell infection.

In this study, we isolated two murine NAbs, 8A9 and 8C12, from gB-immunized mice by hybridoma technology. Both 8A9 and 8C12 showed better neutralizing capacity than AMMO5 by completely neutralizing B-cell infection. 8A9 and 8C12 recognized novel epitopes located on D-II of gB, which was distinct from that of AMMO5. 8A9 and 8C12 exhibited different cross-reactivities with rhLCV gB even though they showed epitope competition. We identified the precise epitope and key residues recognized by AMMO5 for the first time. 8A9, 8C12, and AMMO5 could significantly inhibit gB-mediated membrane fusion. The characterization of 8A9, 8C12, and AMMO5 reveals the key role of the stem region, especially D-II, of gB in eliciting NAbs.

## Materials and Methods

### Cells and Virus

All cell lines were cultured and routinely maintained at 37°C in humidified air containing 5% CO_2_. MDCK cells and 293T cells were maintained in Dulbecco’s modified Eagle medium (DMEM) containing 10% fetal bovine serum (FBS). EBV-negative Akata cells, Sp2/0 cells, and hybridoma cells were grown in RPMI 1640 supplemented with 10% FBS. 293F cells were cultured in OPM-293 CD05 Medium (OPM Biosciences).

CNE2-EBV-GFP cells were induced by 12-*O*-tetradecanoylphorbol 13-acetate (TPA) (20 ng/ml) and sodium butyrate (2.5 mM) for 12 h and then the medium was replaced by RPMI 1640 with 10% FBS. After 72 h, the cultures were clarified by centrifugation at 1,000 *g* for 5 min and then the supernatant was filtrated through 0.45-μm filters. Viruses were concentrated 100-fold by centrifugation at 50,000 *g* for 2.5 h, and the virus pellets were resuspended by RPMI 1640 without FBS. The viruses were stored at −80°C for use.

### Construction and Expression of Protein

The coding sequence of the EBV gB ectodomain (residues 23–683) (UniProt ID: P03188) was cloned with residues ^112^WY^113^ and ^193^WLIW^196^ replaced by HSV-1 residues ^177^HR^178^ and ^258^RVEA^261^, respectively. The coding sequence of the rhLCV gB ectodomain (residues 23–685) (UniProt ID: Q8UZD5) was synthesized with residues ^112^WY^113^ and ^193^WILW^196^ replaced in the same way as for EBV gB. Both gB genes were cloned into pCDNA3.1 with a 6 × His tag at the C-terminus. The plasmids coding for gB domains were constructed by inserting the appropriate DNA fragment into the vectors pCDNA3.1 and pTO-T7-HBc149 (49). The D-I coding sequence (aa 89–294), D-II coding sequence (aa 77–88 and aa 295–390), D-III coding sequence (aa 52–68, aa 455–527, and aa 617–624), D-IV coding sequence (aa 42–51 and aa 528–616), and D-V coding sequence (aa 625–679) were inserted into pCDNA3.1 and pTO-T7-HBc149. The D-I coding sequence (aa 89–294), truncated D-II coding sequence (aa 295–390), truncated D-III coding sequence (aa 455–527), truncated D-IV coding sequence (aa 528–616), and D-V coding sequence (aa 625–679) were inserted into pTO-T7-HBc149.

Plasmids encoding gB of EBV and rhLCV, and EBV gB D-I to D-V, were transfected into 293F cells. The supernatant was collected and purified with Ni-NTA resin (Cytiva). Plasmids encoding truncated forms of gB domains were transformed into BL21(DE3) competent bacteria. Protein production was induced by adding isopropyl-β-D-thiogalactoside (IPTG) at a final concentration of 0.2 mM when OD_600_ = 0.8 at 25°C for 6–8 h. The bacteria were collected by centrifugating at 7,000 *g* for 15 min and resuspended by PBS. Then, the supernatant was collected after ultrasonication and centrifugation. The supernatant was heated at 65°C for 30 min and centrifugated at 12,500 rpm for 10 min. The supernatant was further purified by gel filtration using Superdex 200 10/300 GL (Cytiva).

### Antibody Screening and Purification

Eight-week-old female BALB/c mice were immunized with gB (100 μg/mouse) emulsified with Freund’s adjuvant subcutaneously 3 times at 2-week intervals. Two weeks after the final immunization, mice were boosted again. Three days later, spleen cells of immunized mice were collected and fused with mouse myeloma Sp2/0 cells. The hybridomas secreting gB-specific MAbs were sequentially screened by enzyme-linked immunosorbent assay (ELISA) and neutralization assay. The hybridomas were cloned three times *via* limiting dilution, and purified from mouse ascites using protein A affinity chromatography (Cytiva).

293F cells were transfected with plasmids encoding heavy and light chains of AMMO5 (48). AMMO5 was further purified by protein A affinity chromatography (Cytiva).

### Enzyme-Linked Immunosorbent Assay

To assess the binding ability of antibodies or sera, proteins were coated on 96-well ELISA plates with 100 ng/well at 37°C for 2 h. The plates were washed by PBS containing 0.1% v/v Tween-20 (PBST) once and blocked with PBS containing 2% w/v non-fat dry milk (blocking solution) at 37°C for 2 h. The 2-fold serially diluted antibody was added to the wells and incubated at 37°C for 30 min. After washing five times with PBST, 100 µl of horseradish peroxidase (HRP)-conjugated goat anti-mouse IgG (GAM-HRP) buffer or goat anti-human IgG (GAH-HRP) buffer was added to each well and incubated at 37°C for 30 min. After washing five times with PBST, 100 µl of tetramethylbenzidine (TMB) substrate (Wantai BioPharm) was added and incubated at 37°C in the dark for 15 min. The reaction was stopped with a 2 M H_2_SO_4_ solution. Absorbance was measured at 450 nm using a PHOmo microplate reader (Autobio).

To evaluate antibody binding activities with peptides, streptavidin-immobilized plates (Wantai BioPharm) were coated with biotinylated peptides (200 ng/well) at 37°C for 1 h. Antibody at 1 μg/ml was added to the wells and incubated at 37°C for 30 min. After five washes with PBST, 100 µl of GAM-HRP IgG buffer or GAH-HRP buffer was added to each well and incubated at 37°C for 30 min. After five washes, 100 µl of TMB substrate (Wantai BioPharm) was added at 37°C in the dark for 15 min. The reaction was stopped with a 2 M H_2_SO_4_ solution. Absorbance was measured at 450 nm using a PHOmo microplate reader.

### Surface Plasmon Resonance Assay

BIAcore 8k (Cytiva) was used for evaluating the equilibrium dissociation constant (KD values) of the antibodies. The sensor chip CM5 was coated with proteins *via* covalent coupling. A 70-µl solution of a 1:1 (v/v) of N-ethyl-N-(3-diethylarninopropyl) carbodiimide (EDC) and *N*-hydroxysuccinimide (NHS) was mixed to activate the carboxyl groups on the dextran surface. EBV gB was diluted with 10 mM sodium acetate (pH 5.0) to a final concentration of 1.5 µg/ml for coupling. rhLCV gB was diluted with 10 mM sodium acetate (pH 5.0) to a final concentration of 2.5 µg/ml for coupling. In each channel, only flow cell 2 was coated with ligand while flow cell 1 was empty and blocked by ethanolamine as a control. After chip loading, the serially diluted antibody was injected at 30 µl/min for 300 s (association phase), followed by dissociating at 30 µl/min for 600 s (dissociation phase). The results were analyzed by BIAcore Insight Evaluation software, and the curve fitting was performed using a 1:1 binding model.

To assess the epitope competition with 8A9, EBV gB was coated in sensor chip CM5 at 1.5 µg/ml. 8A9 was diluted to 800 nM and injected at 30 µl/min for 300 s, and antibodies (8A9, 8C12, and AMMO5) at 800 nM were injected for 300 s. The epitope competition with AMMO5 was analyzed in the same way.

### SDS-PAGE and Western Blot

The protein samples mixed with loading buffer containing β-mercaptoethanol were heated at 100°C for 10 min. Then, the mixture was loaded into 4%–12% SurePAGE gels (Gen Script) and run at 180 V for 30 min. Proteins were visualized by Coomassie Brilliant Blue staining for 30 min and destained until the background became transparent.

Proteins on the gels were transferred onto polyvinylidene fluoride membranes (Millipore) after SDS-PAGE. The membranes were incubated with a blocking buffer (Wantai BioPharm) at room temperature for 1 h. The membranes were incubated with 8A9, 8C12, and AMMO5, respectively. After five washes with PBST, the membrane was incubated with GAM-HRP or GAH-HRP at room temperature for 30 min. After five washes with PBST, the WesternBright ECL (Advansta) was used for color development.

### Protein Deglycosylation

PNGase F (NEB) was used to release the N-linked glycans. PNGase F (10 µl) was added with 100 µg of protein, and the final sample was prepared as 100 µl. The sample was incubated in a water bath at 37°C for 12 h. The deglycosylated proteins were analyzed using SDS-PAGE.

### Immunofluorescence

The plasmid encoding full-length EBV gB or rhLCV gB was transfected into MDCK cells for 48 h. The transfected cells were seeded on 96-well plates overnight, and then fixed with 4% paraformaldehyde at room temperature for 15 min and washed twice. The cells were incubated with blocking solution (2% BSA in PBS) at 37°C for 2 h and washed with PBST. Antibodies were used for staining by incubation with cells at 4°C overnight. After two washes with PBST, the cells were labeled with donkey anti-mouse AlexaFluor 488 (Molecular Probes) and goat anti-human IgG AlexaFluor 488 (Molecular Probes) at room temperature for 30 min. After two washes with PBST, the cells were stained with DAPI (Invitrogen) for 5 min. The samples were visualized with the Opera Phenix Plus High-Content Screening System (PerkinElmer).

### Flow Cytometry

293T cells were transfected with plasmid encoding the full-length rhLCV gB for 48 h. The transfected cells were trypsinized and resuspended by PBS and prepared with 10^6^ cells per test. The cells were stained with antibodies at 4°C for 30 min. After two washes, 293T cells were incubated with goat anti-mouse IgG BV421 (BioLegend) and anti-human IgG BV421 (BD Biosciences) at 4°C for 30 min in the dark. Stained cells were analyzed by flow cytometry (FCM) on an LSRFortessaX-20 instrument (BD Biosciences) and the data were evaluated using FlowJo software X 10.0.7 (BD Biosciences).

### Transmission Electron Microscopy

The gB proteins were analyzed by negative staining electron microscopy as previously reported (49). Briefly, diluted protein samples were applied to 200-mesh carbon-coated copper grids (Quantifoil) for 5 min and excess solution was removed. After two washes with doubly distilled water, grids were immediately negatively stained for 30 s with freshly filtered 2% phosphotungstic acid (pH 6.4). Grids were examined with a FEI Tecnai T12 TEM (FEI) at an accelerating voltage of 120 kV and photographed at a magnification of 25,000-fold.

### Neutralization Assay on B Cells

For neutralization of B cells, serially diluted antibodies were incubated with 20 µl of CNE2-EBV-GFP at 37°C for 2 h. The mixture was added into 10^4^ Akata cells/well in 96-well plates. The numbers of GFP-positive cells were measured by the LSRFortessaX-20 instrument (BD Biosciences) after incubation with RPMI 1640 containing 10% FBS for 48 h to determine infection rate. Uninfected cells were used as negative control and Akata cells infected with EBV were regarded as infected control. The neutralizing efficiency of antibodies was calculated by the following equation: neutralization efficiency % = [(% of GFP-positive cells in infected control) − (% of GFP-positive cells in the antibody-treated group)]/% GFP-positive cells in infected control.

### High-Performance Size Exclusion Chromatography

To evaluate the purity of gB protein, EBV gB and rhLCV gB proteins were analyzed by an 1120 Compact LC HPLC system (Agilent Technologies) and separated by a TSKgel G3000PW XL column (TOSOH), which was pre-equilibrated with PBS. The flow rate was 0.5 ml/min and protein signal detection was of 280 nm.

### Virus-Free Cell Fusion Assay

To evaluate the cell fusion blocking efficiency of NAbs 8A9, 8C12, and AMMO5, 293T cells were inoculated in 10-cm dishes in DMEM with 10% FBS at a density of 2×10^6^ cells/dish. When reaching 80% confluence, the cells were transfected with 2.5 μg each of pCAGGS-gB, pCAGGS-gH, pCAGGS-gL, and pCAG-T7. 293T cells in another dish were transfected by 10 μg of pT7EMCLuc. Twenty-four hours after transfection, 2×10^5^ cells transfected with plasmids encoding gB, gH, gL, and T7 polymerase were trypsinized and incubated with 0.8 μg of antibodies at 37°C for 30 min. Then, the mixture of cells and antibodies was added with 2×10^5^ cells transfected with pT7EMCLuc in a 24-well plate and cultured for 24 h at 37°C in DMEM with 10% FBS. Cells were lysed and luciferase activity was quantified using Dual-Glo luciferase assay (Promega).

### Statistical Analysis

All statistical analyses were performed using GraphPad Prism. *p*-values were generated by a one-way ANOVA analysis. Data were considered statistically significant at *p < 0.05.

## Results

### Generation and Characterization of gB-Specific Antibodies

**Figure 1:**
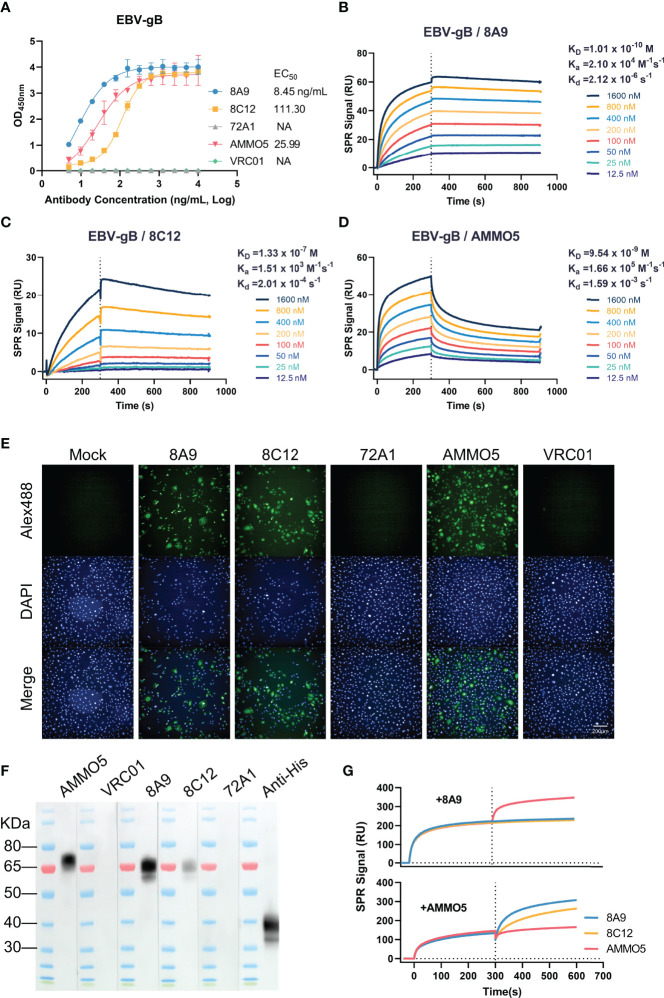
Evaluation of the binding abilities of 8A9, 8C12, and AMMO5. **(A)** The binding abilities of 8A9, 8C12, and AMMO5 against the recombinant gB ectodomain were detected using ELISA. **(B–D)** The binding affinities of gB NAbs against immobilized gB were measured using the SPR assay. **(E)** The reactivities of MAbs with native full-length gB expressed in MDCK cells were measured by the immunofluorescence (IF) assay. **(F)** Detection of reactivities against denatured gB using the Western blot assay. The gp350 antibody 72A1 was used as a negative control for murine antibodies. The HIV-ENV antibody VRC01 was used as the negative control for the human antibody. **(G)** Epitope competition was detected using SPR by preincubating with 8A9 or AMMO5.

gB is the core fusion protein of EBV. We generated two murine antibodies targeting EBV gB, 8A9, and 8C12, from gB-immunized mice. The binding abilities of gB-specific MAbs were evaluated using ELISA and surface plasmon resonance (SPR) assay. 8A9, 8C12, and previously reported NAb AMMO5 (48) showed specific binding against gB with the mean half-maximal effective concentration (EC_50_) value of 8.45, 111.30, and 25.99 ng/ml, respectively (**Figure 1A**). The negative control antibodies gp350 72A1 (50) and HIV-ENV VRC01 (51) did not react with gB (**Figure 1A**).

For SPR analysis, 8A9 exhibited picomolar affinity by following fast-on (association rate constant [*K*_a_] of 2.10 × 10^4^ M^−1^ s^−1^) and slow-off (dissociation rate constant [*K*_d_] of 2.12 × 10^-6^ s^−1^) binding kinetics, resulting in an equilibrium dissociation constant (*K*_D_) of 0.10 nM (**Figure 1B**), which bound 1–3 orders of magnitude more tightly to gB than 8C12 (133.00 nM) and AMMO5 (9.54 nM) (**Figures 1C, D**).

Next, we examined the binding ability of gB MAbs against native protein using immunofluorescence (IF) assay. We found that the three MAbs exhibited specific staining with native full-length gB displayed on the surface of gB-transfected cells, while the negative control antibodies 72A1 and VRC01 had no such effect (**Figure 1E**).

Previous reports demonstrated that some MAbs to gB from the herpesvirus family (HCMV and HSV) recognized linear and continuous epitopes (52, 53). We further characterized the binding ability of 8A9, 8C12, and AMMO5 by Western blot assay, and the result indicated that the three MAbs could react with denatured gB protein (**Figure 1F**), suggesting that their epitopes contained linear regions. According to a previous report, the ectodomain of gB is cleaved into two fragments (~70 kDa and ~40 kDa) under reducing conditions (54). The three MAbs all recognized the slower-migrating fragment (~70 kDa), while the anti-his-tag antibody recognized the faster-migrating fragment (~40 kDa) since the his-tag was fused to the C-terminus of gB (**Figure 1F**). Compared to 8A9 and AMMO5, 8C12 had weaker reactivity with denatured gB, which may be due to the lower binding affinity of 8C12 (**Figures 1A, C**), or indicated that the 8C12 epitope may be partially sensitive to denaturation.

AMMO5 was the only published NAb targeting gB, while its neutralizing epitope on gB had not been reported (48). To evaluate the epitope differences between these three MAbs, we carried out a competition experiment using the SPR assay. We detected an increase in response unit (RU) of AMMO5 when gB was preincubated with 8A9 on the chip (**Figure 1G**), indicating that 8A9 and AMMO5 recognized distinct epitopes. In addition, an excess of 8A9 completely inhibited both 8A9 and 8C12 binding to immobilized gB (**Figure 1G**), suggesting that the epitope of 8A9 may overlap with that of 8C12. Preincubation of AMMO5 did not disturb the binding of 8A9 and 8C12 (**Figure 1G**), indicating that the epitope of 8C12 differed with that of AMMO5.

**Figure 2:**
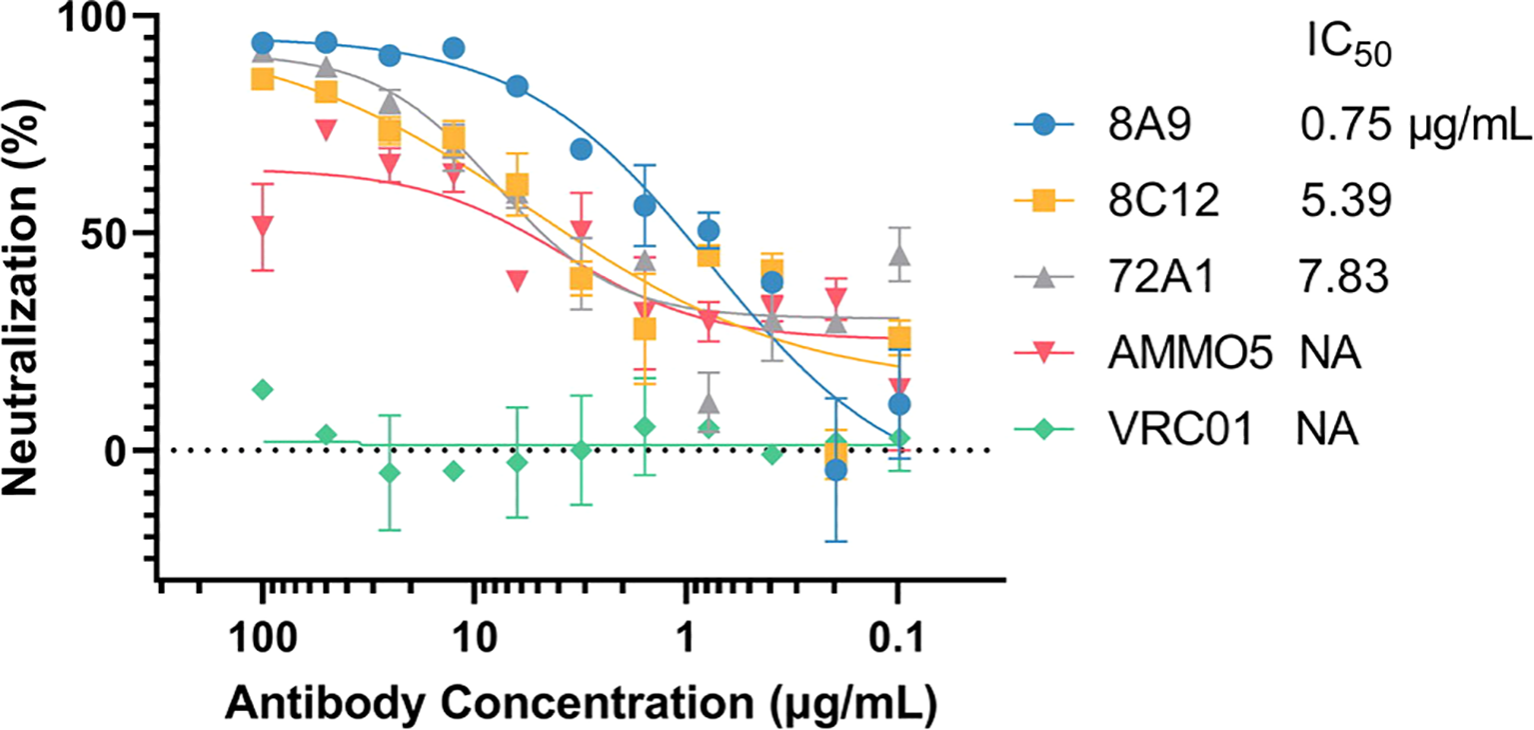
Evaluation of the neutralizing abilities of 8A9, 8C12, and AMMO5. The neutralizing abilities of MAbs were measured in the B-cell infection assay. The MAbs were serially diluted and the IC_50_ was calculated. VRC01 was used as the negative control.

Next, we evaluated the neutralizing ability of 8A9 and 8C12 in a B-cell infection assay. For comparison, we also included gp350 murine NAb 72A1 and gB NAb AMMO5. VRC01 was used as a negative control. Consistent with a previous report (48), AMMO5 could only partially neutralize EBV infection of B cells and reduced B-cell infection to 30% (**Figure 2**). 8A9, 8C12, and 72A1 were capable of completely neutralizing B-cell infection (**Figure 2**). 8A9 showed potent neutralizing capacity with a half maximal inhibitory concentration (IC_50_) value of 0.75 μg/ml, compared to that of 8C12 (5.39 μg/ml) and 72A1 (7.83 μg/ml). 8A9 and 8C12 were the first reported gB NAbs that completely neutralized B-cell infection. Collectively, we had generated two gB antibodies, 8A9 and 8C12, which showed a specific binding ability against recombinant gB and native gB. 8A9 and 8C12 recognized novel epitopes that were different from that of AMMO5. Both 8A9 and 8C12 were capable of neutralizing B-cell infection completely.

### Cross-Reactivity Analysis of gB MAbs

**Figure 3:**
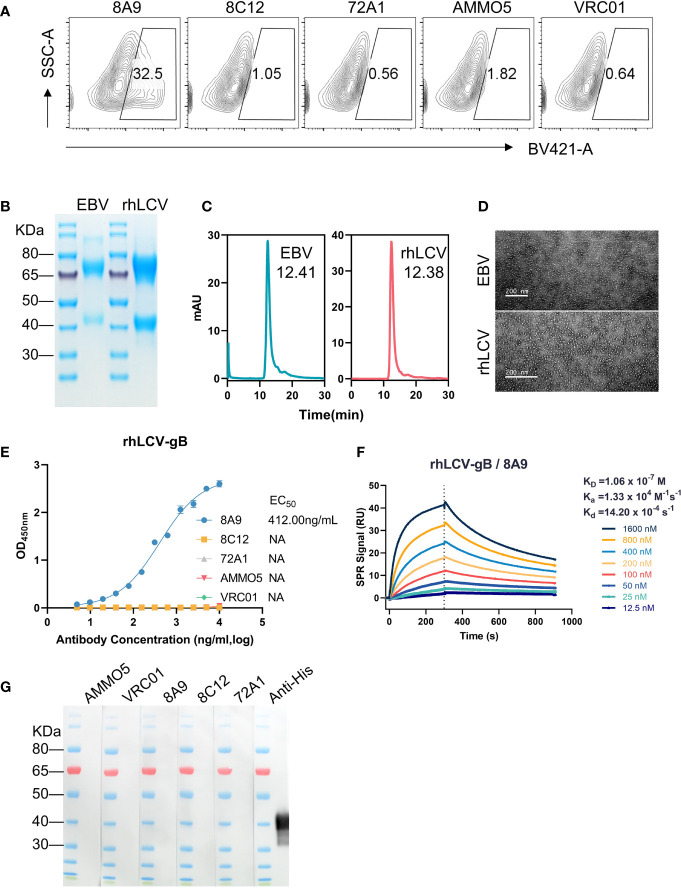
Evaluation of the antibody cross-reactivity with rhLCV gB. **(A)** Evaluation of cross-reactivities of MAbs with native full-length rhLCV gB expressed in 293T cells. The stained cells were analyzed using flow cytometry. **(B)** The SDS-PAGE result of EBV gB and rhLCV gB under reducing conditions. **(C, D)** The size and morphology of gB proteins were evaluated by **(C)** high-performance liquid chromatography (HPSEC) and **(D)** transmission electron microscopy (TEM). **(C)** The retention time was calculated and marked. **(E)** The binding abilities of MAbs with rhLCV gB were measured using ELISA. **(F)** The binding affinity of 8A9 with rhLCV gB was evaluated using the SPR assay. **(G)** Western blot analysis of MAbs with denatured rhLCV gB.

As members of the lymphocryptovirus genus, EBV and its rhesus viral homolog, rhesus lymphocryptovirus (rhLCV), share a high degree of sequence identity (55, 56). rhLCV infection in rhesus macaques serves as a model system for studying EBV infection in human (57–60). To investigate the antibody cross-reactivity, 293T cells transfected with full-length rhLCV gB were stained with gB MAbs and control antibodies (72A1 and VRC01), and the stained cells were analyzed using the FCM assay. The FCM result demonstrated that only 8A9 had cross-reactivity against rhLCV gB while 8C12 and AMMO5 failed to recognize rhLCV gB (**Figure 3A**). A soluble form of rhLCV gB was needed for further assessment of binding activity of 8A9 against rhLCV gB. Similar to EBV gB, rhLCV gB had hydrophobic segments close to the C-terminus (**Figure S1**) (54). We constructed an ectodomain truncation containing residues 23 to 685 of rhLCV gB, which was made just before the beginning of hydrophobic regions in order to avoid the membrane anchor. The ectodomain of wild-type EBV gB had been reported to aggregate since the putative fusion loop regions were composed of highly hydrophobic residues, and resulted in rosette formation (54). The substitution of EBV gB fusion loops with HSV-1 gB fusion loops successfully prevented protein aggregation and allowed the production of separate trimeric gB (54). The putative fusion loops of rhLCV gB (^112^WY^113^ and ^193^WILW^196^) are also hydrophobic and almost identical to that of EBV gB (^112^WY^113^ and ^193^WLIW^196^) (**Figure S1**). To avoid the potential protein aggregation, analogous sequence replacements were constructed by replacing wild-type fusion loops of rhLCV gB with corresponding residues of HSV-1 gB (^177^HR^178^ and ^259^RVEA^261^). The ectodomain variant of rhLCV gB was constructed and successfully expressed using 293F cells. rhLCV gB contains a furin protease cleavage recognition sequence (RRRRR) that is identical to EBV gB (**Figure S1**). The purified rhLCV gB protein migrated as two distinct bands with an apparent molecular mass of ~75 kDa and ~40 kDa under reducing conditions compared to that of EBV gB (~70 kDa and ~40 kDa) (**Figure 3B**). To assess the oligomerization state of rhLCV gB in solution, high-performance size-exclusion chromatography (HPSEC) and native-stained transmission electron microscopy (TEM) analysis were carried out. rhLCV gB showed a single major peak in the HPSEC profiles at a retention time of 12.38 min, shifting a little earlier than EBV gB (12.41 min) (**Figure 3C**). As shown in **Figure 3D**, samples collected from the peak fractions of EBV gB and rhLCV gB both exhibited high purity and trimeric form without rosette formation.

The ELISA result revealed that 8A9 was the only gB antibody that specifically bound to rhLCV gB (**Figure 3E**), consistent with the FCM result (**Figure 3A**). Furthermore, the binding ability of 8A9 against rhLCV gB (EC_50_ = 412.00 ng/ml) was ~50-fold weaker than that against EBV gB (EC_50_ = 8.45 ng/ml). The binding affinity of 8A9 with rhLCV gB (*K*_D_ = 106.00 nM) (**Figure 3F**) was 3 orders of magnitude weaker than that with EBV gB (*K*_D_ = 0.101 nM) (**Figure 1B**). Considering that 8A9 was capable of binding to EBV gB under reducing conditions (**Figure 1F**), we performed Western blot to see whether 8A9 could bind to rhLCV gB in the same condition. Unexpectedly, no obvious reaction was observed from 8A9 against denatured rhLCV gB. 8C12 and AMMO5 did not react with denatured rhLCV gB as expected. Taken together, 8A9 was the first EBV gB NAb that cross-reacted with rhLCV gB, and 8A9 showed weaker binding affinity with rhLCV gB compared to that with EBV gB.

### Epitope Definition of 8A9, 8C12, and AMMO5

Identification and characterization of neutralizing epitopes are of great value to elucidate the neutralizing mechanism of NAbs (61), while no neutralizing epitopes on EBV gB has been reported so far. 8A9, 8C12, and AMMO5 were capable of recognizing denatured EBV gB protein (**Figure 1F**), indicating that their epitopes contained linear and continuous sequences. In order to further determine the specific epitopes, we constructed an overlapping peptide library based on the EBV gB sequence for ELISA analysis. Peptides were 20 amino acids long (10-amino-acid overlap) and named P1 to P66 in order, covering the EBV gB ectodomain (aa 23–692) (**Table S1**). Peptides were conjugated with biotin at the N-terminus, and then immobilized on streptavidin-coated ELISA plates.

**Figure 4:**
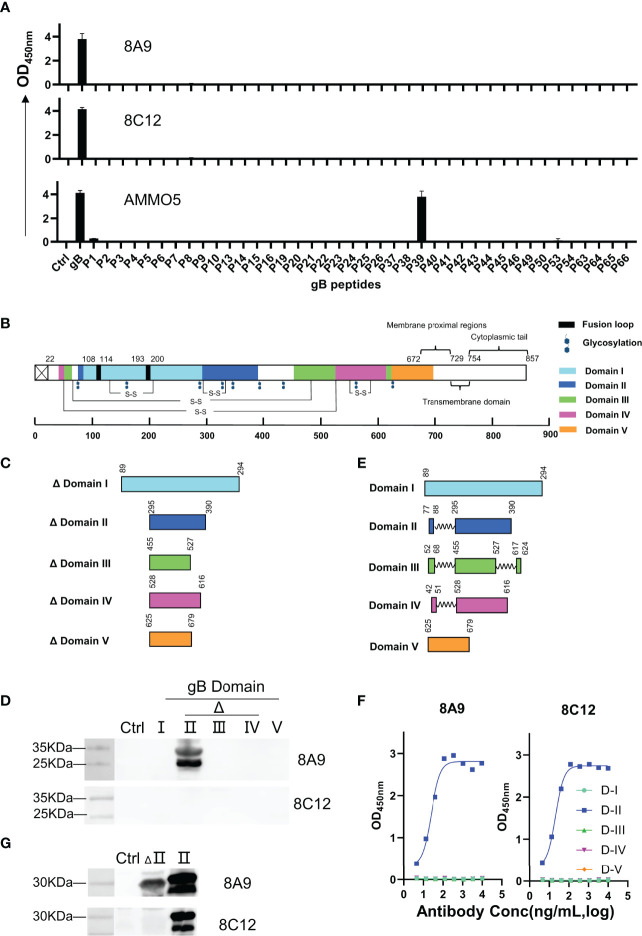
Epitope identification of 8A9, 8C12, and AMMO5. **(A)** Epitope mapping using a gB truncated peptide library covering gB ectodomain (aa 23–692). Each peptide was 20 amino acids long with a 10-amino-acid overlap in the peptide library. **(B)** Schematic representation of gB domains, fusion loops, and putative glycosylation sites located on EBV gB. Fusion loop is colored in black. Glycosylation sites were marked as hexagons. gB D-I to D-V were colored cyan, blue, green, magenta, and orange, respectively. **(C, E)** Graphical representation of **(C)** truncated domains and **(E)** complete domains. **(D)** Western blot analysis of 8A9 and 8C12 for their reactivities with truncated gB domains expressed by *E. coli*. HBc149 vector was used as control. **(F)** ELISA analysis of 8A9 and 8C12 for their activities with purified complete gB domains expressed by 293F cells. **(G)** 8A9 and 8C12 were tested by Western blot assay for reactivity against truncated and complete D-II. HBc149 vector was used as control.

AMMO5 showed strong reaction with P39 (^403^TSSPPSSPSPPAPSAARGST^422^) (**Figure 4A**). Even though the peptide length was 20 amino acids, and the offset number was 10 amino acids, 8A9 and 8C12 showed no reaction with peptides in the peptide library, suggesting that their epitopes may be longer than 20 amino acids or incomplete in the peptide library. To map the epitopes of 8A9 and 8C12, it was needed to evaluate their binding activity with truncated gB. The EBV gB ectodomain is divided into 5 domains and several domains are composed of discontinuous regions (D-II, D-III, and D-IV) (26) (**Figure 4B**). The major and continuous fragment of each domain was chosen and expressed using the prokaryotic expression system (**Figure 4C**). As shown in the Western blot result of **Figure 4D**, 8A9 strongly reacted with truncated D-II (aa 295–390) while 8C12 had no reaction with these domains (**Figure 4D**).

The reactivity of 8C12 with denatured EBV gB was weaker than that of 8A9 (**Figure 1F**), indicating that 8C12 epitope may be sensitive to reducing condition. In addition, the domains used for evaluation were incomplete and lacked partial peptides (**Figure 3C**), which may cause the loss of the epitope needed for 8C12 binding. In order to determine the 8C12 epitope, we constructed complete EBV gB domains by connecting different regions of each domain with a flexible linker (**Figure 4E**). The new constructions of gB domains were expressed in a eukaryotic expression system to retain the native conformation and potential post-translational modification required for antibody binding. The binding activity of 8A9 and 8C12 against different domains was evaluated using ELISA without denaturation of protein. Similarly, 8A9 specifically bound to D-II, consistent with the Western blot result (**Figures 4D, F**). Surprisingly, 8C12 reacted with the complete form of D-II, which contained aa 77–88 and aa 295–390 (**Figure 4F**). We further constructed the complete version of D-II and expressed it using the prokaryotic expression system. The reactivity of 8A9 and 8C12 against two forms of D-II was assessed in Western blot assay. As shown in **Figure 4G**, 8A9 recognized both truncated and complete D-II, and 8C12 could only bind to complete D-II as expected. We found that residues 77–88 (^77^HTEGLLMVFKDN^88^) were included in P6 (^73^TRENHTEGLLMVFKDNIIPY^92^) of the overlapping peptide library (**Table S1**). 8C12 did not react with P6 but recognized complete D-II, indicating that its epitope was discontinuous and located on both aa 77–88 and aa 295–390. To sum up, we confirmed that both 8A9 and 8C12 bound to D-II of gB, and the neutralizing epitope of AMMO5 was identified as well.

### Identification of Key Residues Recognized by AMMO5

**Figure 5:**
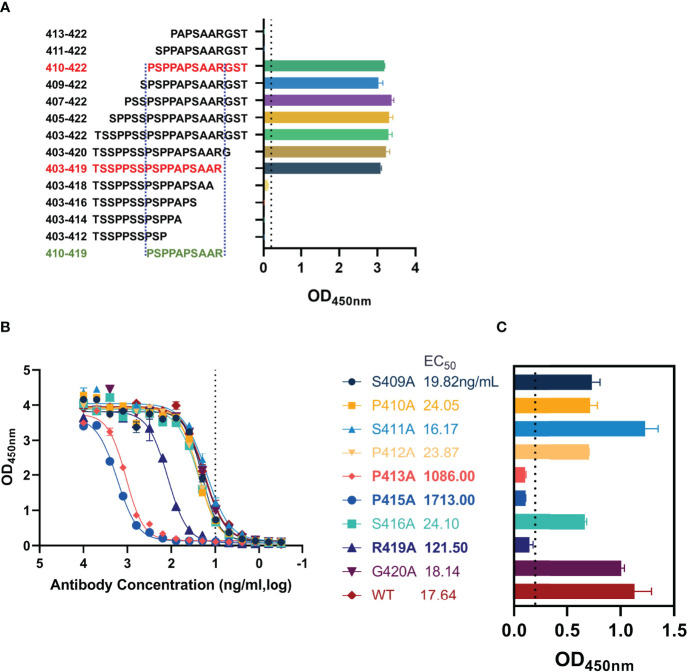
Key residues validation of AMMO5. **(A)** Biotinylated truncated peptides covering aa 403–422 were used to identify the core epitope recognized by AMMO5. **(B, C)** Alanine scanning library spanning aa 410–419 was used for determining the key residues of AMMO5. **(B)** AMMO5 was serially diluted and evaluated using ELISA. **(C)** The OD values were displayed when the antibody concentration was 1 μg/ml.

**Figure 6:**
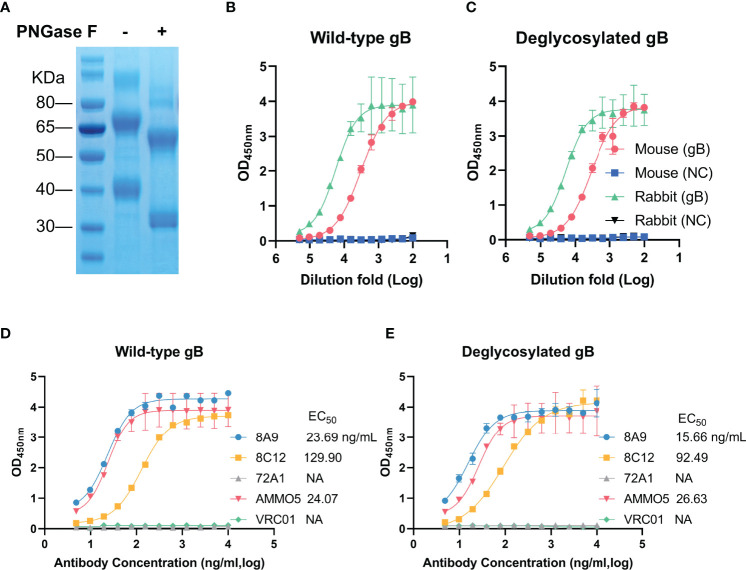
Evaluation of binding abilities with deglycosylated gB of gB NAbs. **(A)** Wild-type (left) and deglycosylated gB (right) were analyzed by SDS-PAGE. **(B, C)** Detection of reactivities of gB-immunized sera with **(B)** wild-type and **(C)** deglycosylated gB. **(D, E)** Evaluation of binding activities of MAbs with **(D)** wild-type and **(E)** deglycosylated gB.

The epitope of AMMO5 was mapped to aa 403–422. To further identify the core region recognized by AMMO5, we constructed a panel of truncated peptides that covered aa 403–422 and carried different lengths of truncation. The binding activity of AMMO5 with truncated peptides was evaluated using ELISA. As shown in **Figure 5A**, peptides with N-terminal truncation to Ser411 or C-terminal truncation to Ala418 showed significant loss of antibody binding (**Figure 6A**), which narrowed down the core epitope of AMMO5 to aa 410–419 of gB. Peptide ^410^PSPPAPSAAR^419^ was the shortest amino acid sequence that retained the binding activity of AMMO5. To identify the key residues located on the epitope of AMMO5, a panel of single alanine substitution peptides spanning aa 410–419 was used to detect the critical residues contributing to antibody binding (residues 414, 417, and 418 were alanine in the wild-type sequence). Alanine mutations to Pro413, Pro415, and Arg419 resulted in diminished binding activity with 1–2 orders of magnitude decrease in EC_50_ values (wild-type: 17.64 ng/ml, P413A: 1,086.00 ng/ml, P415A: 1,713 ng/ml, and R419A: 121.50 ng/ml) (**Figure 5B**). Moreover, the OD values of P413A, P415A, and R419A were less than 0.2 when the antibody concentration was 10 ng/ml (**Figure 5C**). Taken together, we had identified the key residues recognized by AMMO5 as Pro413, Pro415, and Arg419.

### The Binding Abilities of 8A9 and 8C12 Are Not Glycosylation-Dependent

The glycosylation of viral glycoproteins plays an essential role in mediating protein folding, interaction with host receptor, and immune evasion (62). EBV gB has 9 predicted N-linked glycosylation sites (N76, N163, N290, N329, N348, N395, N436, N563, and N629) (26), which are distributed on the surface of EBV gB protein (**Figure 4B**). Two glycosylation sites (N329 and N348) are mapped to D-II and three glycosylation sites (N76, N290, and N395) are located near D-II. To study the relationship between glycosylation and antibody binding ability, we used an endoglycosidase, PNGase F, to specifically remove N-linked glycans from EBV gB. The addition of PNGase F caused a significant reduction in molecular weight (two bands at ~60 kDa and ~32 kDa) of deglycosylated gB compared to that of wild-type gB (two bands at ~70 kDa and ~40 kDa) (**Figure 6A**), which was consistent with a previous report (63). We next evaluated the antigenicity of the deglycosylated EBV gB with sera from gB-immunized rabbits and mice. Immunization sera showed equivalent reactivities with deglycosylated gB and wild-type gB (**Figures 6B, C**), suggesting that deglycosylation did not affect overall structure and conformational epitopes of gB, and deglycosylated gB retained native antigenicity. Furthermore, we evaluated the binding abilities of 8A9, 8C12, and AMMO5 against deglycosylated gB and wild-type gB. As shown in **Figures 6D, E**, deglycosylation did not interfere with the binding activities of antibodies since they showed comparable EC_50_ value against wild-type gB (8A9: 23.69 ng/ml, 8C12: 129.90 ng/ml, and AMMO5: 24.07 ng/ml) and deglycosylated gB (8A9: 15.66 ng/ml, 8C12: 92.49 ng/ml, and AMMO5: 26.63 ng/ml). The data suggested that the epitopes of 8A9, 8C12, and AMMO5 were not glycosylation-dependent.

### 8A9 and 8C12 Inhibit Membrane Fusion

**Figure 7:**
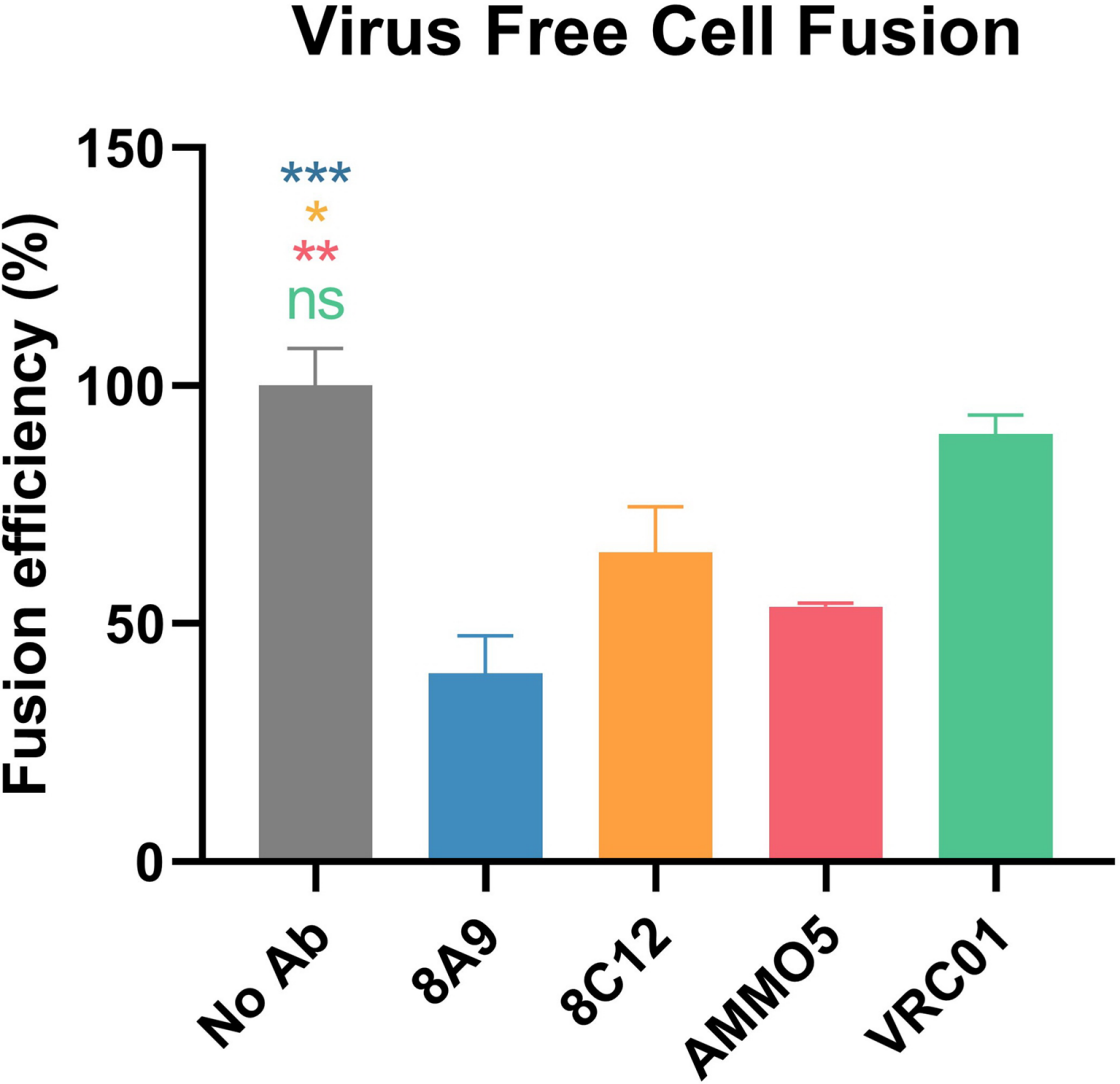
Inhibition of cell–cell fusion by gB NAbs. Evaluation of gB NAbs using a virus-free cell fusion assay. Relative fusion efficiency was calculated in percentage terms. All data are presented as mean ± SEM. *p < 0.05; **p < 0.01; ***p < 0.001; ns, not significant.

EBV mainly infects epithelial cells and B cells by different routes (18). The process of infection is complex, and composed of various steps, including viral attachment and membrane fusion (14, 64). Distinct combinations of glycoproteins are required during EBV infection across different cell types (65). gB is the viral fusion protein that is involved in EBV infection of both epithelial cells and B cells, and is also the final executor of fusion between viral envelope and host cell membrane (64). To further identify whether 8A9 and 8C12 could inhibit membrane fusion, a virus-free cell fusion assay was performed (48, 66). AMMO5 was reported to be capable of reducing cell fusion (48) and was used as a positive control. We observed that 8A9, 8C12, and AMMO5 all significantly inhibited cell–cell fusion, whereas the negative control VRC01 failed to do so (**Figure 7**). 8A9, 8C12, and AMMO5 reduced membrane fusion efficiency to 39%, 65%, and 54%, respectively (**Figure 7**). In summary, 8A9, 8C12, and AMMO5 were able to block membrane fusion even if they recognized different epitopes.

## Discussion

Several EBV NAbs have shown protection in mice and non-human primates (57, 67, 68), while no gB NAbs has been evaluated for protection efficiency *in vivo*. Most animal models for EBV infection are mainly based on infection of B lymphocytes, and associated with the formation of lymphomas (12, 23, 44, 57, 69). Hence, NAbs that effectively inhibit B-cell infection are believed to perform better with *in vivo* models (67). AMMO5 is the only reported gB NAb, while AMMO5 failed to completely block EBV infection in B cells (48). In our study, 8A9 and 8C12 were capable of neutralizing B-cell infection (**Figure 2**), and may provide better protection and therapeutic potential against EBV challenge *in vivo*. The identification of 8A9, 8C12, and AMMO5 indicates that gB is another principal target for induction of potent NAbs. gB is a promising new candidate for therapeutic agents or prophylactic vaccines against EBV infection.

In the present study, we have mapped the epitopes of newly identified 8A9 and 8C12 to regions on D-II, which were distinct from the AMMO5 epitope (gB aa 410–419) (**Figures 4** and **5**). Based on the homology modeling result (**Figures S2A, B**), we found that D-II and AMMO5 binding sites were accessible on the surfaces of both postfusion and prefusion of gB, suggesting that each epitope of 8A9, 8C12, and AMMO5 may be similar or even identical to the two conformations of gB. In addition, we found that the epitopes of gB NAbs were within the binding site of NRP1 (**Figure S2C**). This suggests that 8A9, 8C12 and AMMO5 may prevent gB-induced membrane fusion by limiting EBV gB binding to its co-receptor. High-resolution structures of immune complexes consisting of antibody and gB are required to illustrate the neutralizing mechanism differences of 8A9, 8C12, and AMMO5. The AMMO5 epitope was unresolved in the EBV gB structure due to its flexibility (26), while we supposed that AMMO5 could be included into D-II since its sequence and possible position were close to D-II (**Figures S1** and **S2**). Linker insertion mutations within D-II significantly reduced the gB-derived fusion efficiency, indicating that D-II is essential for membrane fusion (70). In addition, the three antibodies significantly inhibited membrane fusion (**Figure 7**), and their epitopes were located to D-II, indicating that D-II was a vulnerable site for NAb development.

rhLCV is closely related to EBV, and an rhLCV model provides an ideal surrogate for studying EBV infection (55–57, 59, 60). In this study, we found that 8A9 showed cross-reactivity with rhLCV gB, whereas 8C12 and AMMO5 had no such effect (**Figure 3A**). The identification of 8C12 may help to better understand gB functions during lymphocryptovirus infection. However, 8A9 was unable to react with denatured rhLCV gB even if 8A9 recognized a linear epitope on EBV gB (**Figure 3G**), suggesting that 8A9 may bind to the two forms of gB in different ways. In addition, the cross-reactivity with rhLCV gB may enable 8A9 to be further evaluated in a rhesus macaque model. However, it is necessary to identify whether 8A9 can neutralize rhLCV infection *in vitro* firstly. Moreover, the construction of a humanized 8A9 is required to avoid human anti-murine antibody (HAMA) response. Affinity maturation is also needed since 8A9 only showed modest binding affinity with rhLCV gB (**Figure 3F**).

Glycosylation is closely associated with functions of EBV glycoproteins (71–74). gp350 is an extensively glycosylated protein, and the high glycosylation density on gp350 helps to stabilize its conformation and retain antigenicity and immunogenicity (71, 72). The glycosylation is involved in membrane fusion, and the mutations on gL glycosylation sites affect the fusion efficiency (74). gp150 serves as an immunoevasin by forming a glycan shield to prevent antigen presentation (73). Little is known about the relationship between glycosylation and EBV gB function (26). However, N-linked glycans on HSV-2 gB are proven to be essential for gB intracellular trafficking, protein maturation, membrane fusion, and viral entry (75). We sought to identify the influence for EBV gB caused by deglycosylation, and found that deglycosylated gB exhibited similar antigenicity compared to wild-type gB (**Figures 6B, C**). In addition, EBV gB NAbs showed similar binding potency with two forms of gB (**Figures 6D, E**), indicating that the antibody binding was not glycan-dependent. However, the major antigen domains of HCMV gB are surrounded by glycosylation sites, suggesting that the neutralizing epitopes may be shielded by glycans (36). Taken together, more investigations are needed to identify the potential functions of glycans on EBV gB.

In summary, we reported two gB NAbs 8A9 and 8C12, which completely neutralized EBV infection in B cells compared to AMMO5. 8A9, 8C12, and AMMO5 recognized recombinant and native forms of gB with high affinity while only 8A9 cross-reacted with rhLCV gB. The binding abilities of 8A9, 8C12, and AMMO5 were not glycosylation-dependent. The epitopes of 8A9 and 8C12 were mapped to D-II, and the precise epitope of AMMO5 was identified. 8A9, 8C12, and AMMO5 were capable of effectively inhibiting membrane fusion. The identification of 8A9 and 8C12 provides a new insight into the rational design of therapeutics or vaccines focusing on D-II of gB.

## Data Availability Statement

The datasets presented in this study can be found in online repositories. The names of the repository/repositories and accession number(s) can be found in the article/**Supplementary Material**

## Ethics Statement

The animal study was reviewed and approved by the Ethics Committee of Xiamen University Laboratory Animal Center.

## Author Contributions

JH, NX, XZ, and YC designed the studies. JH, DW, LZ, QW, KC, WZ, and YY performed experiments. JH, DW, LZ, QW, and JC analyzed the data. JH, XZ, and YC wrote the paper. All authors contributed to the article and approved the submitted version.

## Funding

The study was supported by grants from the National Natural Science Foundation of China (grant 82073756) and XMU Training Program of Innovation and Enterpreneurship for Undergraduates.

## Conflict of Interest

The authors declare that the research was conducted in the absence of any commercial or financial relationships that could be construed as a potential conflict of interest.

## Publisher’s Note

All claims expressed in this article are solely those of the authors and do not necessarily represent those of their affiliated organizations, or those of the publisher, the editors and the reviewers. Any product that may be evaluated in this article, or claim that may be made by its manufacturer, is not guaranteed or endorsed by the publisher.
