# Supplementary material for: Glycoprotein B Antibodies Completely Neutralize EBV Infection of B Cells
Source: Front Immunol. 2022 May 27;13:920467. doi: 10.3389/fimmu.2022.920467 (PMC9197244; doi:10.3389/fimmu.2022.920467)
Supplement: Supplementary file 1 [file DataSheet_1.docx]

Supplementary Material

| **No.** | **Sequence** | **No.** | **Sequence** |
| --- | --- | --- | --- |
| P1 | QTPEQPAPPATTVQPTATRQ | P34 | EKYEAVQDRYTKGQEAITYF |
| P2 | TTVQPTATRQQTSFPFRVCE | P35 | TKGQEAITYFITSGGLLLAW |
| P3 | QTSFPFRVCELSSHGDLFRF | P36 | ITSGGLLLAWLPLTPRSLAT |
| P4 | LSSHGDLFRFSSDIQCPSFG | P37 | LPLTPRSLATVKNLTELTTP |
| P5 | SSDIQCPSFGTRENHTEGLL | P38 | VKNLTELTTPTSSPPSSPSP |
| P6 | TRENHTEGLLMVFKDNIIPY | P39 | TSSPPSSPSPPAPSAARGST |
| P7 | MVFKDNIIPYSFKVRSYTKI | P40 | PAPSAARGSTPAAVLRRRRR |
| P8 | SFKVRSYTKIVTNILIYNGW | P41 | PAAVLRRRRRDAGNATTPVP |
| P9 | VTNILIYNGWYADSVTNRHE | P42 | DAGNATTPVPPTAPGKSLGT |
| P10 | YADSVTNRHEEKFSVDSYET | P43 | PTAPGKSLGTLNNPATVQIQ |
| P11 | EKFSVDSYETDQMDTIYQCY | P44 | LNNPATVQIQFAYDSLRRQI |
| P12 | DQMDTIYQCYNAVKMTKDGL | P45 | FAYDSLRRQINRMLGDLARA |
| P13 | NAVKMTKDGLTRVYVDRDGV | P46 | NRMLGDLARAWCLEQKRQNM |
| P14 | TRVYVDRDGVNITVNLKPTG | P47 | WCLEQKRQNMVLRELTKINP |
| P15 | NITVNLKPTGGLANGVRRYA | P48 | VLRELTKINPTTVMSSIYGK |
| P16 | GLANGVRRYASQTELYDAPG | P49 | TTVMSSIYGKAVAAKRLGDV |
| P17 | SQTELYDAPGWLIWTYRTRT | P50 | AVAAKRLGDVISVSQCVPVN |
| P18 | WLIWTYRTRTTVNCLITDMM | P51 | ISVSQCVPVNQATVTLRKSM |
| P19 | TVNCLITDMMAKSNSPFDFF | P52 | QATVTLRKSMRVPGSETMCY |
| P20 | AKSNSPFDFFVTTTGQTVEM | P53 | RVPGSETMCYSRPLVSFSFI |
| P21 | VTTTGQTVEMSPFYDGKNKE | P54 | SRPLVSFSFINDTKTYEGQL |
| P22 | SPFYDGKNKETFHERADSFH | P55 | NDTKTYEGQLGTDNEIFLTK |
| P23 | TFHERADSFHVRTNYKIVDY | P56 | GTDNEIFLTKKMTEVCQATS |
| P24 | VRTNYKIVDYDNRGTNPQGE | P57 | KMTEVCQATSQYYFQSGNEI |
| P25 | DNRGTNPQGERRAFLDKGTY | P58 | QYYFQSGNEIHVYNDYHHFK |
| P26 | RRAFLDKGTYTLSWKLENRT | P59 | HVYNDYHHFKTIELDGIATL |
| P27 | TLSWKLENRTAYCPLQHWQT | P60 | TIELDGIATLQTFISLNTSL |
| P28 | AYCPLQHWQTFDSTIATETG | P61 | QTFISLNTSLIENIDFASLE |
| P29 | FDSTIATETGKSIHFVTDEG | P62 | IENIDFASLELYSRDEQRAS |
| P30 | KSIHFVTDEGTSSFVTNTTV | P63 | LYSRDEQRASNVFDLEGIFR |
| P31 | TSSFVTNTTVGIELPDAFKC | P64 | NVFDLEGIFREYNFQAQNIA |
| P32 | GIELPDAFKCIEEQVNKTMH | P65 | EYNFQAQNIAGLRKDLDNAV |
| P33 | IEEQVNKTMHEKYEAVQDRY | P66 | GLRKDLDNAVSNGRNQFVDG |

**Supplementary Table 1.** **The summary of peptide sequences of gB truncation peptide library.**





**Supplementary Figure 1.** **Alignment of EBV and rhLCV gB.**

Alignment of EBV (Uniprot ID: P03188) and rhLCV (Uniprot ID: Q8UZD5) gB using ESPript 3.0. The identical amino acids were marked in red color.


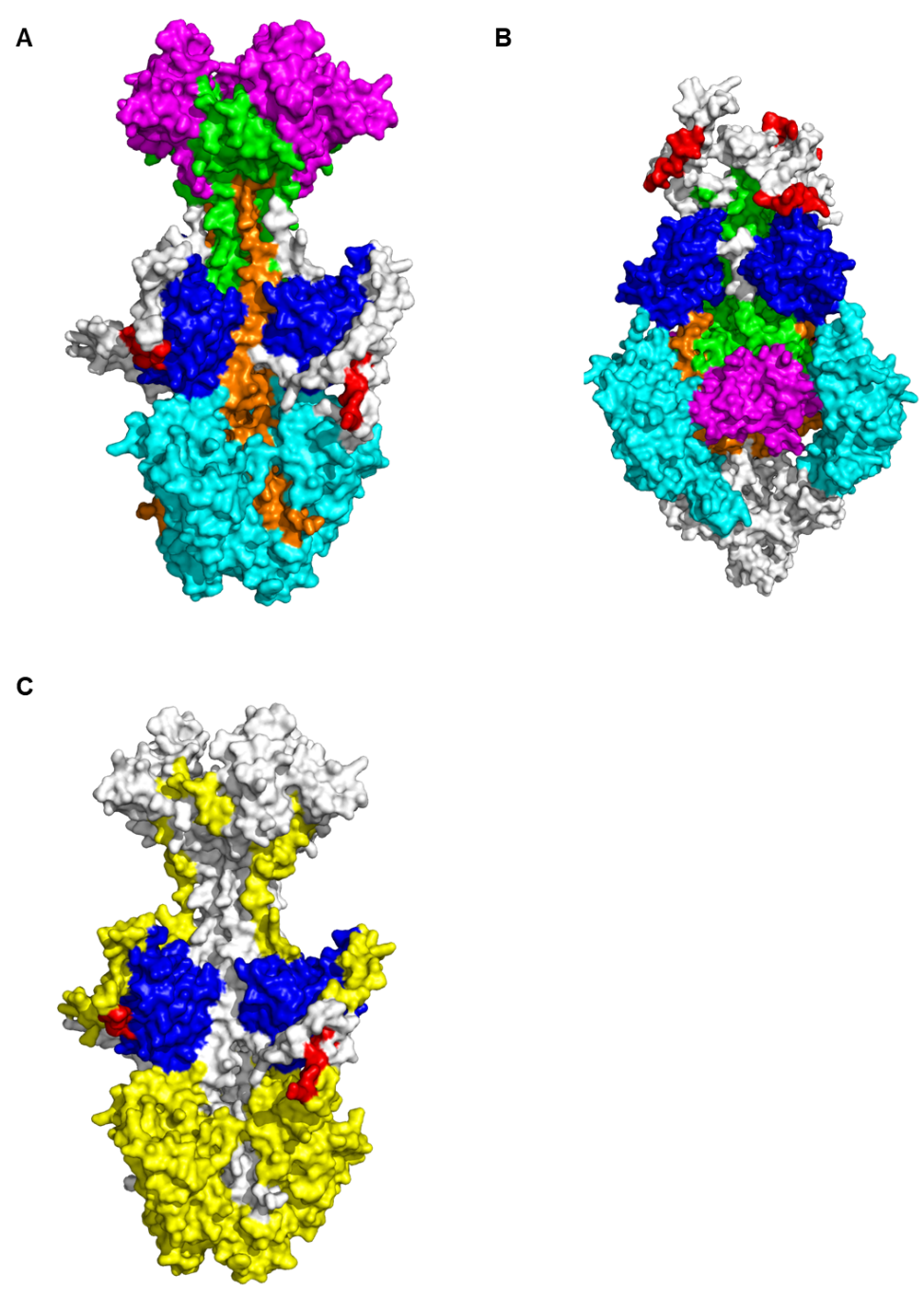


**Supplementary Figure 2.** **Graphical representation of gB domains, AMMO5 binding site and NRP1 binding site.**

(A and B) Different gB domains and AMMO5 binding site were displayed on (A) post-gB and (B) pre-gB based on the result of homology modeling. gB D-I to D-V were colored in cyan, blue, green, magentas and orange, respectively. AMMO5 binding site was marked in red. (C) The NRP1 binding site, AMMO5 binding site and gB D-II were displayed on post-gB. The NRP1 binding site, AMMO5 binding site and gB D-II were colored in yellow, red and blue, respectively. Post-gB structure was the homology modeling result based on the template of EBV gB structure (PDB ID: 3FVC). Pre-gB structure was the homology modeling result based on the template of HCMV pre-gB (PDB ID: 7KDP). The homology modeling was performed using SWISS-MODEL. Images were edited with PyMOL.
